# Dental caries experience among qat chewers in dental college clinics, Jazan, Saudi Arabia

**DOI:** 10.1186/s12903-023-02804-7

**Published:** 2023-02-20

**Authors:** Esam Halboub, Abdulwahab Ahmed Alamri, Ghazi Kamili, Ahmed Shaher Alqahtani

**Affiliations:** 1 Department of Oral and Maxillofacial Surgery and Diagnostics Sciences, College of Dentistry, Jazan University, Jazan, Saudi Arabia; 2 Dental Intern, Internship Program, College of Dentistry, Jazan University, Jazan, Saudi Arabia

**Keywords:** Qat chewing, Dental caries, Saudi Arabia

## Abstract

**Aims:**

There is a considerable debate on the impact of qat chewing on dental and oral health. Thereby, this study aimed at assessing the dental caries among qat chewers (QC) and non-qat chewers (NQC) who attend outpatient dental clinics, College of Dentistry, Jazan, Saudi Arabia.

**Materials and methods:**

A sample of 100 QC and 100 NQC were recruited amongst those attended dental clinics, college of dentistry, Jazan University during the 2018–2019 academic year. Their dental health was assessed by three pre-calibrated male interns using DMFT index. The Care Index, Restorative Index and Treatment Index were calculated. Comparisons between both subgroups were done using independent *t* test. Multiple linear regression analyses were further conducted to stand on the independent determinants of the oral health among this population.

**Results:**

QC were unintentionally older (36.55 ± 8.74 years) than NQC (32.96 ± 8.49 years; *P* = 0.004). Fifty-six percent of QC reported brushing their teeth compared to only 35% (*P* = 0.001). NQC with university and postgraduate educational levels were more than QC. The mean Decayed [5.91 (5.16)] and DMFT [9.15 (5.87)] were higher among QC compared to NQC [3.73 (3.62) and 6.7 (4.58); *P* = 0.001 and 0.001, respectively]. The other indices were not different between both subgroups. The multiple linear regression revealed that qat chewing and age, individually or together, as independent determinants for the dental decay, missing, DMFT and TI.

**Conclusion:**

Qat chewing habit has a detrimental impact on dental health. It is associated with higher dental caries and missing teeth, and a lower treatment index.

## Introduction

Qat, also known as khat, chat, miraa, murungu and Arabian or Abyssinian tea, is an evergreen shrub belongs to the Celastraceae family, and scientifically called Catha edulis [1]. It is cultivated, and even wildly grows, in some African countries and southwest of the Arabian Peninsula [1, 2]. The habit of qat chewing is a deep-rooted cultural habit in these areas [3]. Qat chewing is known to produce an amphetamine-like stimulating effect, and that is why it is habitually chewed [4]. The session of qat chewing lasts several hours. Briefly, the fresh leaves and twigs of qat are chewed to form a bolus which kept on one side of the mouth and continually chewed to release the qat extract which swallowed and/or absorbed through oral mucosa [3, 5–8]. Studies estimated that up to 49% of males in Jazan, a region in the southeast of Saudi Arabia, practice the habit, and the figure increases to 62% in Jazan rural areas [9–13]. Qat chewing is reportedly to have negative systemic effects [14]. In line with that, qat chewing is claimed to exert deteriorating effects on oral soft and hard tissues [15, 16]. However, the reports linking qat chewing with negative health effects are conflicting.

One example of these deteriorating effects of qat chewing is dental caries—the most prevalent, multifactorial, chronic human disease [17]. Although it is mainly an infectious disease, many overlapping and interacting-with each other predisposing factors play a role. Habits, whether good or bad, are essential components of these factors. Qat chewing, smoking, smokeless tobacco use, vaping,…etc. are examples of the bad habits reported to predispose to dental caries. However, evidence on the potential association between qat chewing and dental caries is lacking owing to the conflicting results obtained by the previous studies [17–22]. Therefore, this study sought to assess the potential association of qat chewing with dental caries in a sample of Jazan population who attend outpatient dental clinics, College of Dentistry, Jazan, Saudi Arabia.

## Materials and methods

This cross sectional study was conducted during the first and second semesters of the 2018–2019 academic year. The study protocol was approved by the Scientific Research Committee, College of Dentistry, Jazan University, Saudi Arabia (CODJU-181291), and was conducted with strict adherence to The Declaration of Helsinki on Medical Research involving Human Subjects [23].

The population frame was all dental patients who attended the outpatient dental clinics during the above mentioned period. The study aimed to include a sample of qat chewers (QC) and non qat chewers (NQC) provided that they were 20–50 years old and medically fit males. Being medically fit was confirmed via self-reported medical questionnaire, and an interview, electronic-based, dentist-administered questionnaire; both are routinely used in the college. With regard to QC group, they had to chew qat for at least 5 years, 3–7 days a week, and at least 2 h each time in order to be defined as QC. Smokeless tobacco users were decided to be excluded in order to minimize the cofounding factors. The sample size was calculated using G*power program Version 3.1.9.6 to detect a clinically significant difference in DMFT score between QC and NQC. The following scenario was proposed: DMFT score for QC and NQC are 6 ± 4 and 4.5 ± 3 (the difference = 1.5), and the values of significance and power of this two-tailed scenario were set at 0.05 and 0.8, respectively. The yielded total sample was 178 divided into two equal groups (QC and NQC, 89 each). For more precision, we included 200 subjects (100 QC and 100 NQC).

The clinical examination was done by three pre-calibrated examiners in outpatient clinics on dental chair using the dental examination set. The Decayed, Missing, and Filled Teeth (DMFT) index was used to determine the dental status. Clinical training on scoring the teeth based on DMFT was conducted by a specialist (EH). Then calibration was done on ten subjects (not included in the sample) to make sure the three examiners had the necessary skill to detect the caries and correctly record the DMFT index. Once the three examiners achieved perfect agreement (Kappa = 1), they proceeded to collect data for the main study. In brief, the teeth were washed with water spray with using suction, and dried with air spray and cleaned with gauze. Then DMFT was determined for each tooth and recorded into a specialized form included with an interview form which was designed to collect—in addition to DMFT—information on age, place of residence (rural or urban), education level, and oral hygiene practices. The form also covered the personal history of qat chewing (years since commencing chewing habit, and frequency of chewing in term of days per week, and hours per session). The Care Index (CI = [F/D + M + F] × 100), Restorative Index (RI = ([F/D + F] × 100) and Treatment Index ([TI = F + M/D + M + F] × 100) were calculated [24].

Data were obtained as exel file from which it was imported into SPSS software version 21 (IBM Corp., Armonk, NY, USA) which was used for statistical analyses. Differences in the demographic data and oral health practices, if present, were assessed using Fisher’s exact test or Chi-square test as appropriate. The outcome variables (DMFT with its individual components, CI, RI, and TI) were assessed for normal distribution using Kolmogorov–Smirnov test. As the data were normally distributed, comparisons between both subgroups were done using parametric test (independent *t* test). Multiple linear regression analyses were further conducted to stand on the independent determinants of these outcomes among the study sample. The regression analyses were adjusted for age, residency area, education and tooth brushing and flossing.

## Results

The QC were slightly older (36.55 ± 8.74 years) than NQC (32.96 ± 8.49 years; *P* = 0.004). QC and NQC were equally distributed by residency area (urban versus rural; *P* = 0.5) but not equally distributed according to the education levels: NQC with university and postgraduate educational levels were more than QC (35% versus 14%; *P* = 0.001). Similarly, more NQC reported brushing their teeth than QC did (56% versus 35%; *P* = 0.004). However, no statistical difference was found regarding dental flossing (11% of NQC 5% of QC; *P* = 191). Most of the QC reported practicing the habit for > 5–15 years (36%) and > 15 years (38%) since onset of the habit, for > 2–4 h (30%) and > 4 h (38%) per a session, and > 3 times a week (61%). Fifty-seven of the QC (57%) reported chewing in the left side (Table 1).

**Table 1:** Demographic and qat chewing characteristics of the study sample

Variables	Qat chewers; n (%)	Control (non-qat chewers); n (%)	*P* value^a^
*Residency*			
Urban	41 (41)	40 (40)	0.5
Rural	59 (59)	60 (60)	
*Education*			
Illiterate	3 (3)	4 (4)	0.001
Less than high school	31 (31)	14 (14)	
High school	52 (52)	47 (47)	
University	14 (14)	33 (33)	
Postgraduate	0 (0)	2 (2)	
*Tooth brushing*			
Yes	35 (35)	56 (56)	0.004
No	65 (65)	44 (44)	
*Flossing*			
Yes	5 (5)	11 (11)	0.191
No	95 (95)	89 (89)	
*Chewing time*			
5–10 years	26 (26)	NA	NA
> 10–15 years	36 (36)	NA	
> 15 years	38 (38)	NA	
*Chewing period*			
2 h	32 (32)	NA	NA
> 2–4 h	30 (30)	NA	
> 4 h	38 (38)	NA	
*Chewing frequency*			
2 times per week	26 (26)	NA	NA
3 times per week	13 (13)	NA	
> 3 times per week	61 (61)	NA	
*Chewing side*			
Right	43 (43)	NA	NA
Left	57 (57)	NA	
Age^b^; *mean* ± *SD*	36.55 ± 8.74	32.96 ± 8.49	0.004

*NA* not applicable

^a^Fisher’s exact test or Chi-square test

^b^Independent t test

With regard to the dental caries experience, DMFT was statistically higher among QC (9.15 ± 5.87) compared to NQC (6.7 ± 4.58; *P* = 0.001). Most of the fraction of the DMFT index was attributed to the D component, which was statistically higher among QC compared to NQC (5.91 ± 5.16 and 3.73 ± 3.62, respectively, *P* = 0.001). The M and F components were not statistically different between groups (*P* = 0.111 and 0.267, respectively. Similarly, were the CI, RI, and TI (*P* = 0.08, 0.101, 0.081, respectively). Table 2 provides more details.

**Table 2:** DMFT, its individual components, CI, RI, and TI by qat chewing

Variables	Qat chewers; *mean (SD)*	Control (non-qat chewers); *mean (SD)*	*P* value^a^
Decay	5.91 (5.16)	3.73 (3.62)	0.001
Missing	1.67 (3.62)	1 (2.09)	0.111
Filling	1.57 (2.32)	1.97 (2.83)	0.276
DMFT	9.15 (5.87)	6.7 (4.58)	0.001
CI (%)	20.41 (28.32)	28.48 (34.62)	0.08
RI (%)	23.85 (32.11)	32.19 (37.13)	0.101
TI (%)	34.36 (32.64)	43.17 (36.37)	0.081

*CI* care index, *RI* restorative index, *TI* treatment index (see the text for more details)

^a^Independent *t* test

Table 3 presents the result of the multiple linear regression analyses which revealed that qat chewing and age, individually or together, were the independent determinants for the dental Decay, Missing, DMFT and TI. Qat chewing explained 5.2% of the variability of the D component (R^2^ = 0.052; *P* < 0.001), and 7.2% when combined with age (R^2^ = 0.072; *P* = 0.012). Qat chewing and age together explained 10.2% of the variability of the TI (R^2^ = 0.102; *P* = 0.009): qat chewing alone explained 2.8%, leaving 7.2% for age (R^2^ = 0.072; *P* < 0.001). Qat chewing explained 4.7% of the variability of the DMFT (R^2^ = 0.047; *P* = 0.001), while the age alone explained 14.6% of the M component (R^2^ = 0.146; *P* < 0.001).

**Table 3:** Independent determinants of DMFT, its individual components, CI, RI, and TI

Dependent variable	Determinants	B (95% CI)	Adjusted R^2^	*P* value
Decay	Qat chewing	− 2.51 (− 3.76 to − 1.26)	0.052	< 0.001
	Age	− 0.09 (− 0.16 to − 0.02)	0.072	0.012
Missing	Age	0.131 (0.087–0.175)	0.146	< 0.001
DMFT	Qat chewing	− 2.45 (− 3.92 to − 0.982)	0.047	0.001
TI	Age	1.235 (0.692–0.1.777)	0.074	< 0.001
	Qat chewing	12.874 (3.288–22.46)	0.102	0.009

*TI* treatment index (see the text for more details), *B* unstandardized beta (beta coefficient). The regression analyses were adjusted for age, residency area, education, and tooth brushing and flossing

## Discussion

There is growing evidence links qat chewing with many systemic and oral diseases. However, the evidence on the habit association with development of dental caries is conflicting, with many study supported this notion [18, 19, 25–27] while others didn’t [15, 20, 21]. In light of such a controversy, this study aimed to fill the gap. The results, based on the bivariate and multivariate analyses, support the notion that qat chewing is associated with higher dental caries experience. It must be emphasized that the multivariate statistical model adjusted for the other confounding factors like education and oral health behaviors, a matter which ensures revealing only the effects of the real factors, which were age and qat chewing in our study. Collectively, this confirms that qat chewing diminishes the potential positive effects of university and postgraduate educational levels and the good oral health practices. In our study, the education was statistically significant in the univariate analysis, and this is highly supported by many previous studies [21, 28–30]. Similarly, the univariate analysis revealed that the tooth brushing was significantly more among NQC than QC. Although it is a foregone conclusion that good oral heath practices are protective against dental caries [31, 32], the multivariate analysis didn’t support this in our study. Taken together confirms, at least theoretically, that qat chewing negatively nullifies the positive effects of these factors. Overall, the study provides significant results require a serious attitude and prompt intervention from the authorities.

How qat chewing exerts such deleterious effects is unknown: whether it is due to the chemical composition of qat, mechanical irritation, and/or indirect modifying effects on the positive factors is a hot topic for future research. What matters more, however, is how to tackle this habit. In other meaning how to persuade the current QC to give up and prevent NQC indulging into the habit.

Up to 65% and 56% of DMFT scores among QC and NQC were attributed to the Decay (D) component. This reflects the extent to which that the included subjects, more specifically QC, were indifferent regarding seeking dental treatment. This is confirmed more through the Missing (M) component, which was significantly higher among QC, and through Filling (F) component, which was higher among NQC, although insignificant. Further confirmation comes from the care, restorative, and treatment indices (CI, RI, and TI) which were, although insignificantly, higher among NQC, reflecting they had sought treatment more frequently than QC did.

Although the statistical models revealed significant roles for qat chewing and age, individually or together, the explained variabilities remain small, ranging from 4.7 to 14.6%, indicative of existence of other factors, not included in the study, play roles. Indeed, dental caries is a multifactorial disease where so many biological, microbiological, environmental, socioeconomic, and behavioral factors interact with each to ultimately cause dental caries [33, 34]. However, we have to confirm that we included two major factors which are well known to be associated with dental caries as indicated above: education, and oral health behaviors, namely toothbrushing and flossing. Irrespective they were significant determinants of dental caries in univariate analysis, they lost their effects upon statistical adjusting with multivariate analysis, suggestive they are confounding factors dominated by qat chewing and age. This may mean either many of the respondents, more specifically QC, misleadingly reported that they had university and postgraduate educational levels, and they used to practice positive oral health practices in order to reflect positive image on their selves, a matter which may contaminate the results, or that qat chewing exerts highly negative effects that surpass, and even diminish, the positive effects of these factors.

The study has a few limitations worthy to mention. First, the sample size was small, although we had calculated it in advance, and we could find both statistical and clinical differences, meaning that larger sample was not required to achieve the same results, although undoubtedly would have been better, and we recommend it for future research. Second, the study didn’t include females due to difficulties in finding females who admit chewing qat. Based on consistent observations in Jazan, socially, qat chewing is unacceptable habit, and those who indulged in practicing it try to hide. This applies more to women who less practice this habit in general. Third, the age of the subjects was restricted to 20–50 years. However, we aimed to lessen the effects of age and associated systemic diseases on the oral cavity. Worthy to note that all included subjects were medically fit. Fourth, the gingival/periodontal health was not assessed, although this would have been essential given the conflicting results with which the medical literature is saturated. Fifth, caries detection was done clinically, while early proximal caries can’t be confirmed without intraoral (bitewing) radiographs. But as we adopted clinical examination to measure DMFT in both groups, this doesn’t represent a major breach in the research methodology. However, we recommend taking intraoral radiograph as adjunct to clinical examination in similar future research. Finally, the dental examination was done by three dental interns, a matter which may raise doubt regarding the reliability of measurement. However, we confirm that the examiners were well trained and pre-calibrated. They only commenced examination once they obtained similar results from 10 subjects examined under supervision for the purpose of calibration.

## Conclusion

To sum up, qat chewing habit has a detrimental impact on dental health. More specifically, it is associated with higher dental caries and missing teeth, and a lower treatment index.

## Data Availability

The corresponding author confirms that the data will be made available upon justified request.
